# *LAG3/CD4* Genes Variants and the Risk for Restless Legs Syndrome

**DOI:** 10.3390/ijms232314795

**Published:** 2022-11-26

**Authors:** Félix Javier Jiménez-Jiménez, Javier Gómez-Tabales, Hortensia Alonso-Navarro, Christopher Rodríguez, Laura Turpín-Fenoll, Jorge Millán-Pascual, Ignacio Álvarez, Pau Pastor, Marisol Calleja, Rafael García-Ruiz, Santiago Navarro-Muñoz, Marta Recio-Bermejo, José Francisco Plaza-Nieto, Esteban García-Albea, Elena García-Martín, José A. G. Agúndez

**Affiliations:** 1 Section of Neurology, Hospital Universitario del Sureste, E28500 Arganda del Rey, Spain; 2 ARADyAL Instituto de Salud Carlos III, University Institute of Molecular Pathology Biomarkers, Universidad de Extremadura, E10003 Cáceres, Spain; 3 Section of Neurology, Hospital La Mancha-Centro, E13600 Alcázar de San Juan, Spain; 4 Fundació per la Recerca Biomèdica i Social Mútua de Terrassa, E08221 Terrassa, Spain; 5 Movement Disorders Unit, Department of Neurology, Hospital Universitari Mutua de Terrassa, E08221 Terrassa, Spain; 6 Unit of Neurodegenerative Diseases, Department of Neurology, The Germans Trias i Pujol Research Institute (IGTP), University Hospital Germans Trias i Pujol, E08916 Badalona, Spain; 7 Department of Medicine-Neurology, Universidad de Alcalá, E28801 Alcalá de Henares, Spain

**Keywords:** restless legs syndrome, genetics, genetic polymorphisms, LAG3 gene, CD4 gene, risk factors

## Abstract

According to several studies, inflammatory factors could be related to the pathogenesis of idiopathic restless legs syndrome (RLS). In addition, RLS and Parkinson’s disease (PD) have shown a possible relationship, and recent studies have shown an association between *CD4* rs1922452 and *CD4* rs951818 single nucleotide variants (SNVs) and the risk for PD. For these reasons, we investigated the possible association between common variants in the *LAG3/CD4* genes (which encoded proteins involved in inflammatory and autoimmune responses) and the risk for RLS in a Caucasian Spanish population. We assessed the frequencies of *CD4* rs1922452, *CD4* rs951818, and *LAG3* rs870849 genotypes and allelic variants in 285 patients with idiopathic RLS and 350 healthy controls using a specific *TaqMan*-based qPCR assay. We also analyzed the possible influence of the genotypes’ frequencies on several variables, including age at onset of RLS, gender, family history of RLS, and response to drugs commonly used in the treatment of RLS. We found a lack of association between the frequencies of genotypes and allelic variants of the 3 SNVs studied and the risk of RLS, and a weak though significant association between the *CD4* rs1922452 GG genotype and an older age at onset of RLS. With the exception of this association, our findings suggest that common SNVs in the *CD4/LAG3* genes are not associated with the risk of developing idiopathic RLS in Caucasian Spanish people.

## 1. Introduction

Restless legs syndrome (RLS), also known as Willis–Ekbom disease (WED), is a neurological disorder with a high prevalence [1], characterized mainly by sensory-motor symptoms, whose diagnostic criteria have been well established [2]. Although much evidence suggests an important role of genetic factors in the etiology of RLS, the causative genes have not yet been fully identified. While initial Genomic Wide Association Studies (GWAS) identified 6 susceptibility genes, further GWAS and meta-analysis identified a total of 21 susceptibility loci, in addition to confirming the 6 previously described [3,4,5]. However, taken together, these loci of susceptibility would account for only 11.3% of the heritability of RLS [4].

The neurochemical characteristics of RLS are also not fully known, although the most important are dopaminergic dysfunction and iron deficiency, and a possible contribution of other neurotransmitter systems (the GABAergic, glutamatergic and adenosinergic systems, at least) has been described [6]. Several recent findings suggest a role of inflammatory factors in the etiopathogenesis of RLS:

(A) RLS is associated to several inflammatory and autoimmune diseases, including chronic liver disease [7], Sjogren’s syndrome [7], rheumatoid arthritis [7], sarcoidosis [7], systemic lupus erythematous [7], inflammatory bowel disease [7,8], multiple sclerosis [9], mastocytosis [10], and chronic obstructive pulmonary disease [11].
(B) There is a description of patients suffering from recurrent and severe RLS always coinciding with infectious-inflammatory conditions [12].
(C) There is a description of higher serum/plasma levels of C reactive protein (CRP) or high-specific CRP (hsCRP) in patients with idiopathic RLS (iRLS) compared with controls, according to several studies [13,14] but not to others [15,16,17].
(D) There is an association between increased serum/plasma hsCRP levels and odds for severe periodic leg movements of sleep (PLMS) in untreated patients with RLS [18].
(E) There is a description of significantly higher serum/plasma levels of hsCRP in patients with end-stage kidney disease with RLS compared with those without RLS, and correlation between hsCRP and RLS severity, according to one study [19]. Another three studies in patients with chronic kidney disease did not show significant differences in serum/plasma hsCRP between patients with and without RLS [20,21,22]. Mandal et al. [11] showed a lack of significant differences in serum/plasma hsCRP levels in patients with chronic obstructive pulmonary disease with and without RLS.
(F) There is a description of a higher neutrophil-to-lymphocyte ratio (NLR), a marker of systemic inflammation, in patients with RLS than in controls in a cross-sectional [23] and a prospective longitudinal study [14], although in the latter the statistical significance disappeared after adjusting for sex, age, body mass index and smoking status [14].
(G) There is a description of higher serum interleukin 1β (IL-1β), IL-6, and tumor necrosis factor α (TNF-α) levels in RLS patients than in controls according to one study [16], and of similar plasma cytokine-specific autoantibodies (c-aAb) against IL-1α, IL-6, IL-10, and GM-CSF (granulocyte-macrophage colony-stimulating factor) in patients with RLS and controls, a higher frequency of c-aAb against IFN-α (interferon-alpha) in RLS patients, and significantly higher plasma suPAR (soluble urokinase-type plasminogen activator receptor) levels in patients with severe RLS (although the latter disappeared after adjusting for sex, age, body mass index, and smoking status) in another [14]. IL-6 and TNF-α were not related to the presence of PLMS in RLS patients [18]. Serum IL-6 was increased in patients with end-stage kidney disease and RLS compared with those without RLS [18] and was similar in patients with hematologic malignancies with and without RLS [24].
(H) There is a description of the up-regulation or down-regulation of several proteins involved in inflammatory processes in patients with RLS compared with controls in proteomic studies in serum/plasma [25,26].
(I) There is a description of an association of several polymorphisms in the IL-1β IL-17α genes but of a lack of association of polymorphisms of other IL, IFN, TNF-α, and nuclear factor kappa-B (NFKB) genes with the risk for RLS in patients with HIV infection [27].

Encoded by the *lymphocyte activation gene 3* (*LAG3*) or *CD223* gene (chromosome 12p13.31; MIM 153337m gene ID 3902), LAG3 protein, which is expressed by both activated and exhausted CD4+ and CD8+ T cells, regulatory T cells, and microglia, acts by delivering inhibitory signals that regulate immune cell homeostasis, T cell activation, proliferation, cytokine production, cytolytic activity and other functions related to inflammatory responses [28,29]. The *LAG3* gene is closely related to the *CD4 molecule* gene (*CD4*; MIM 186940; gene ID 920) [30]. This gene encodes the CD4 membrane glycoprotein of T lymphocytes, which is an important mediator of immune and inflammatory responses. Several polymorphisms in the *LAG3* and *CD4* genes have been linked to the severity of primary immune thrombocytopenia (rs870849 T > C) [31], multiple sclerosis (rs1922452 A > G) [32], disease progression, and the mortality of sepsis (rs951818 C > A) [33].

Two recent case-control association studies, one in a Chinese population [34] and the other in a Caucasian Spanish population [35], have shown a weak, though significant, association between *CD4* rs1922452 and *CD4* rs951818 single nucleotide variants (SNVs) and the risk for Parkinson’s disease (PD).

Although RLS and PD have shown significant differences in terms of their clinical, neuroimaging, neuropathological and genetic characteristics, both diseases share some common data, such as a dopaminergic deficiency and response to drugs with dopaminergic action [36]. In addition, a recent meta-analysis has shown a significantly higher prevalence of RLS in patients with PD than in controls [36], and a genetic case-control association study of cases and controls has shown a decreased risk for RLS in carriers of the longest-size-variant allele (allele 2) of the complex microsatellite repeat Rep1 within the *α-synuclein gen* (*SNCA*, *PARK1*) [37]. In this study, we aim to establish whether these polymorphisms associated with the risk for PD are associated with the risk for RLS in Caucasian Spanish people.

## 2. Results

The frequencies of all genotypes and allelic variants, both in RLS and healthy control groups, followed Hardy–Weinberg’s equilibrium and did not differ significantly between both study groups, either in the whole series (Table 1) or when men and females were analyzed separately (Supplementary Tables S1 and S2); nor were they influenced by the positivity of the family history of RLS, with the exception of a significantly lower frequency of the rs1922452AA genotype in patients without a family history of RLS, which disappeared after a correction for multiple comparisons (Table 2). In a stratified analysis classifying RLS patients according to their response to dopaminergic drugs (Supplementary Table S3), clonazepam (Supplementary Table S4), and GABAergic drugs (Supplementary Table S5), we found a statistically significant increased frequency of patients with the rs951818 A/C genotype responding to GABAergic drugs compared to patients lacking a response to these drugs (*p* = 0.013), and an increased frequency of the rs1922452A allele in patients responding to clonazepam in comparison with those not responding to this drug (*p* = 0.034), which also disappeared after a correction for multiple comparisons. The age at the onset of RLS was significantly higher in patients with the rs1922452 GG genotype as compared with the other two genotypes of this SNV (Table 3). The RLS severity (Table 4) did not differ significantly between the different genotypes in RLS patients, except for a higher severity in patients with the rs1922452 GG genotype as compared with the rs1922452 AG genotype.

## 3. Discussion

The possible role of inflammatory factors in the pathogenesis of RLS, suggested by the frequent association of RLS with several inflammatory diseases [7,11], and the increase in inflammatory markers in patients with RLS or iRLS, such as CRP/hsCRP, NLR, and several cytokines found in several (although not all) studies [13,14,15,16,17,18,19,20,21,22,23,24,25,26], makes it reasonable to investigate possible genetic susceptibility factors related to inflammation. In this regard, the proteins encoded by *LAG3/CD4* genes play an important role in inflammatory and immune responses. As previously commented, at least one SNV in these genes has been related to the risk for multiple sclerosis [32], a paradigm of inflammatory and neurodegenerative disease, which is frequently associated with secondary RLS [38]. Moreover, two recent studies have shown an association of two SNVs in the *LAG3/CD4* genes with the risk for PD [34,35]. Based on the previously mentioned possible relationship between PD and RLS [36], we assessed the possible association between these SNVs and the risk for PD.

The results of the current study, which involved Caucasian Spanish people, failed to show any major association of common SNPs between *CD4* rs1922452, *CD4* rs951818, and *LAG3* rs870849 SNVs and the risk of developing RLS. However, individuals carrying the *CD4* rs1922452 AA genotype were more prone to have a negative family history of RLS, and those carrying the *CD4* rs1922452 GG genotype showed more of a trend towards an older age at onset than those carrying the *CD4* rs1922452 AA genotype. Regarding the response to drugs usually used for the treatment of RLS, we identified weak associations between several SNVs and the response to clonazepam and GABAergic drugs, which should be interpreted cautiously (since the frequency of non-responders was somewhat low) and would require further confirmation.

The sample size of the two analyzed cohorts (both RLS patients and controls) constitutes the main limitation of the current study. This sample size, though adequate for the detection of ORs of 1.5, is insufficient to detect more modest associations. Considering this limitation, the current study reports a lack of association between the three most common SNVs in the *LAG3/CD4* genes and the risk of developing RLS in Caucasian Spanish people, although a possible relationship of several SNVs with the age at onset of RLS or with the pharmacological response to certain drugs is modestly suggested. Despite the fact that the main results of this study are “negative” (“negative” results being as valid as “positive” results), it presents a plausible hypothesis and fulfils the proposed standards of validity for studies with negative results, such as reporting statistical power and confidence intervals as well as primary outcomes [39,40]. An alternative hypothesis, such as the possibility that other SNVs in the *LAG3/CD4* gene could be associated with a modification of the risk for RLS, is not precluded by these results.

## 4. Materials and Methods

### 4.1. Patients and Controls

A total of 285 patients with idiopathic RLS (iRLS), according to the International Restless Legs Syndrome Study Group (IRLSSG) diagnostic criteria [2], and 350 age- and sex-matched healthy controls were involved in this study. Approximately 60 percent of patients included in the current study had participated in case-control genetic association studies previously reported by our group [41,42,43,44,45,46,47,48,49,50,51]. The inclusion of patients with iRLS implied the prior exclusion of all those with causes of secondary RLS, as described in detail elsewhere [48]. The recruitment of patients with RLS was done in the movement disorders units of four hospitals, and healthy controls (none of whom had a previous history, personal or family-related, of RLS or other movement disorders) were recruited from students or staff of the University of Extremadura. Clinical and demographic data from both patients with iRLS and controls are summarized in Table 5.

### 4.2. Ethical Aspects

Approval of the study was given by the Ethics Committees of the Hospital La Mancha–Centro (Alcázar de San Juan, Ciudad Real, Spain), University Hospital “Príncipe de Asturias” (LIB 02/2017; Alcalá de Henares, Madrid, Spain), and the University Hospital of Badajoz (Badajoz, Spain). Before inclusion in the study, which was conducted per the principles of the Declaration of Helsinki, participants signed an informed consent form.

### 4.3. Genotyping of CD4 rs1922452, CD4 rs951818, and LAG3 rs870849 Variants

Genomic DNA, which was obtained from peripheral leukocytes of venous blood samples of patients diagnosed with iRLS and controls, was used to perform genotyping studies. To perform the analysis, real-time PCR (Applied Biosystems 7500 qPCR thermocycler) with specific TaqMan probes (Life Technologies, Alcobendas, Madrid, Spain) was used. The single nucleotide variations (SNVs) analyzed in this study were the same as those analyzed in two other case-control studies involving PD patients [34,35], that is, one intronic and one non-coding transcript exonic SNV with high allele frequencies, and the only missense SNVs with an allele frequency over 0.01 in the population analyzed here. The respective SNVs analyzed, and the corresponding TaqMan tests performed, were rs1922452 (C__11914936_10), rs951818 (C___8921385_10), and rs870849 (C___9797874_10).

### 4.4. Statistical Analysis

The statistical analysis was performed using the SPSS 27.0 version for Windows (SPSS Inc., Chicago, IL, USA). The online program https://ihg.gsf.de/cgi-bin/hw/hwa1.pl (last accessed, 31 August 2022) was used to confirm the Hardy–Weinberg equilibrium, both in RLS patients and controls. The chi-square test, or Fisher’s exact test where appropriate, were used to calculate the intergroup comparison values. We calculated the 95% confidence intervals and also the negative predictive values [52], and we performed the correction for multiple comparison adjustments using the False Discovery Rate (FDR) [53].

We used a genetic model to analyze the frequency of the lower allele with an odds ratio (OR) value = 1.5 (α = 0.05) from the allelic frequencies found in healthy subjects to perform the calculation of the sample size. Considering the sample size of this study, the statistical power (two-tailed association) for variant alleles was 94.7% for rs1922452, 94.6% for rs951818, and 94.2% for rs870849.

Finally, a T-test for independent samples was used to perform comparisons of the mean age at the onset of RLS symptoms and the severity of RLS symptoms according to the IRLSSG scale [54] between the different genotypes.

## Figures and Tables

**Table 1:** Genotypes and allelic variants of patients with RLS and healthy volunteers. The values in each cell represent the number (percentage; 95% confidence intervals). P: crude probability; Pc: probability after multiple comparisons; NPV: negative predictive value.

Genotype	RLS Patients (*n* = 285, 570 alleles)	Controls (*n* = 350, 700 alleles)	OR (95% CI), P; Pc; NPV (95% CI)
rs1922452 A/A	52 (18.2; 13.8–22.7)	59 (16.9; 12.9–20.8)	1.10 (0.73–1.66); 0.647; 0.955; 0.56 (0.54–0.57)
rs1922452 A/G	135 (47.4; 41.6–53.2)	165 (47.1; 41.9–52.4)	1.01 (0.74–1.38); 0.955; 0.955; 0.55 (0.51–0.59)
rs1922452 G/G	98 (34.4; 28.9–39.9)	126 (36.0; 31.0–41.0)	0.94 (0.67–1.29); 0.672; 0.955; 0.55 (0.52–0.58)
rs951818 A/A	99 (34.7; 29.2–40.3)	127 (36.3; 31.2–41.3)	0.94 (0.67–1.30); 0.685; 0.955; 0.55 (0.52–0.58)
rs951818 A/C	144 (50.5; 44.7–56.3)	169 (48.3; 43.1–53.5)	1.09 (0.80–1.50); 0.575; 0.955; 0.56 (0.52–0.60)
rs951818 C/C	42 (14.7; 10.6–18.9)	54 (15.4; 11.6–19.2)	0.95 (0.61–1.47); 0.809; 0.955; 0.55 (0.53–0.57)
rs870849 C/C	120 (42.1; 36.4–47.8)	138 (39.4; 34.3–44.5)	1.17 (0.81–1.54); 0.499; 0.955; 0.56 (0.53–0.60)
rs870849 C/T	128 (44.9; 39.1–50.7)	168 (48.0; 42.8–53.2)	0.88 (0.65–1.21); 0.438; 0.955; 0.54 (0.50–0.57)
rs870849 T/T	37 (13.0; 9.1–16.9)	44 (12.6; 9.1–16.0)	1.04 (0.65–1.66); 0.877; 0.955; 0.55 (0.54–0.57)
**Alleles**			
rs1922452 A	239 (41.9; 37.9–46.0)	283 (40.4; 36.8–44.1)	1.06 (0.85–1.33); 0.589; 0.877; 0.56 (0.53–0.58)
rs1922452 G	331 (58.1; 54.0–62.1)	417 (59.6; 55.9–63.2)	0.94 (0.75–1.17); 0.589; 0.877; 0.54 (0.51–0.58)
rs951818 A	342 (60.0; 56.0–64.0)	423 (60.4; 56.8–64.1)	0.98 (0.78–1.23); 0.877; 0.877; 0.55 (0.51–0.58)
rs951818 C	228 (40.0; 36.0–44.0)	277 (39.6; 35.9–43.2)	1.02 (0.81–1.28); 0.877; 0.877; 0.55 (0.53–0.58)
rs870849 C	368 (64.6; 60.6–68.5)	444 (63.4; 59.9–67.0)	1.05 (0.83–1.32); 0.676; 0.877; 0.56 (0.52–0.60)
rs870849 T	202 (35.4; 31.5–39.4)	256 (36.6; 33.0–40.1)	0.95 (0.76–1.20); 0.676; 0.877; 0.55 (0.53–0.57)

**Table 2:** Genotypes and allelic variants of patients with RLS distributed by family history. The values in each cell represent the number (percentage; 95% confidence intervals).

Genotype	Positive Family History of RLS (*N* = 182, 364 ALLELES)	Negative Family History of RLS (*N* = 99, 198 ALLELES)	Intergroup Comparison Values OR (95%CI) P, PC, NPV (95%CI)
rs1922452 A/A	26 (14.3; 9.2–19.4)	25 (25.3; 16.7–33.8)	0.49 (0.27–0.91); 0.023; 0.207; 0.32 (0.29–0.35)
rs1922452 A/G	93 (51.1; 43.8–58.4)	41 (41.4; 31.7–51.1)	1.48 (0.90–2.42); 0.121; 0.363; 0.40 (0.34–0.45)
rs1922452 G/G	63 (34.6; 27.7–41.5)	33 (33.3; 24.0–42.6)	1.06 (0.63–1.78); 0.829; 0.906; 0.36 (0.31–0.40)
rs951818 A/A	68 (37.4; 30.3–44.4)	29 (29.3; 20.3–38.3)	1.44 (0.85–2.44); 0.175; 0.394; 0.38 (0.34–0.42)
rs951818 A/C	85 (46.7; 39.5–54.0)	57 (57.6; 47.8–67.3)	0.65 (0.39–1.06); 0.082; 0.363; 0.30 (0.24–0.36)
rs951818 C/C	29 (15.9; 10.6–21.3)	13 (13.1; 6.5–19.8)	1.25 (0.62–2.54); 0.530; 0.906; 0.36 (0.33–0.38)
rs870849 C/C	76 (41.8; 34.6–48.9)	43 (43.4; 33.7–53.2)	0.93 (0.57–1.53); 0.786; 0.906; 0.35 (0.30–0.40)
rs870849 C/T	83 (45.6; 38.4–52.8)	43 (43.4; 33.7–53.2)	1.09 (0.67–1.79); 0.727; 0.906; 0.36 (0.31–0.41)
rs870849 T/T	23 (12.6; 7.8–17.5)	13 (13.1; 6.5–19.8)	0.96 (0.46–1.98); 0.906; 0.906; 0.35 (0.33–0.37)
**Alleles**			
rs1922452 A	145 (39.8; 34.8–44.9)	91 (46.0; 39.0–52.9)	0.96 (0.46–1.98); 0.160; 0.480; 0.35 (0.33–0.37)
rs1922452 G	219 (60.2; 55.1–65.2)	107 (54.0; 47.1–61.0)	1.28 (0.91–1.82); 0.160; 0.480; 0.39 (0.34–0.43)
rs951818 A	221 (60.7; 55.7–65.7)	115 (58.1; 51.2–65.0)	1.12 (0.78–1.59); 0.543; 0.815; 0.37 (0.32–0.42)
rs951818 C	143 (39.3; 34.3–44.3)	83 (41.9; 35.0–48.8)	0.90 (0.63–1.28); 0.543; 0.815; 0.34 (0.31–0.38)
rs870849 C	235 (64.6; 59.6–69.5)	129 (65.2; 58.5–71.8)	0.97 (0.68–1.40); 0.889; 0.889; 0.35 (0.29–0.41)
rs870849 T	129 (35.4; 30.5–40.4)	69 (34.8; 28.2–41.5)	1.03 (0.71–1.48); 0.889; 0.889; 0.35 (0.32–0.38)

**Table 3:** Age at onset of RLS according to the genotypes.

	Age at Onset (SD) Years	Two-Tailed *t*-Test Compared to A/A	Two-Tailed *t*-Test Compared to A/G
rs1922452 AA	40.13 (18.09)		
rs1922452 AG	42.52 (16.71)	0.394	
rs1922452 GG	46.28 (18.85)	0.038	0.069
		**Two-Tailed *t*-Test compared to A/A**	**Two-Tailed *t*-Test compared to A/C**
rs951818 AA	42.86 (18.02)		
rs951818 AC	44.75 (17.24)	0.413	
rs951818 CC	41.19 (19.44)	0.624	0.256
		**Two-Tailed *t*-Test compared to C/C**	**Two-Tailed *t*-Test compared to C/T**
rs870849 CC	44.79 (17.89)		
rs870849 CT	43.27 (18.13)	0.507	
rs870849 TT	40.47 (16.64)	0.199	0.407

*t*-Test rs1922452 AA vs. the rest of genotypes: *p* = 0.126.

**Table 4:** Severity of RLS (IRLSSG) according to the genotypes.

	IRLSSG (SD)	Two-Tailed *t*-Test Compared to A/A	Two-Tailed *t*-Test Compared to A/G
rs1922452 AA	24.91 (6.80)		
rs1922452 AG	23.27 (6.72)	0.157	
rs1922452 GG	26.10 (6.38)	0.316	0.002
		**Two-Tailed *t*-Test compared to A/A**	**Two-Tailed *t*-Test compared to A/C**
rs951818 AA	25.39 (5.94)		
rs951818 AC	24.11 (6.86)	0.144	
rs951818 CC	23.90 (7.85)	0.235	0.870
		**Two-Tailed *t*-Test compared to C/C**	**Two-Tailed *t*-Test compared to C/T**
rs870849 CC	24.50 (5.32)		
rs870849 CT	24.55 (7.53)	0.950	
rs870849 TT	24.43 (7.96)	0.951	0.932

**Table 5:** Demographic and clinical data of the series studied.

Group	Controls (*n* = 350)	RLS (*n* = 285)
**Age, y, mean (SD)**	55.7 (16.3)	56.1 (14.9)
**Age at onset, y, mean (SD)**	NA	43.7 (17.6)
**Female %**	270 (77.1%)	217 (77.1%)
**Positive family history %**	NA	190 (66.6%) ^1^
**IRLSSG, mean (SD)**	NA	24.74 (6.39)

^1^ Family history was recorded for 281 out of the 285 patients.

## Data Availability

All data related to the current study, intended for reasonable use, is available from J.A.G. Agúndez (University Institute of Molecular Pathology Biomarkers, University of Extremadura—UNEx ARADyAL Instituto de Salud Carlos III, Av/ de la Universidad S/N, E10071 Cáceres. Spain) and F.J. Jiménez–Jiménez (Section of Neurology, Hospital del Sureste, Arganda del Rey, Madrid, Spain).

## Supplementary Materials

The supporting information can be downloaded at: https://www.mdpi.com/article/10.3390/ijms232314795/s1.
