# Supplementary material for: LAG3/CD4 Genes Variants and the Risk for Restless Legs Syndrome
Source: Int J Mol Sci. 2022 Nov 26;23(23):14795. doi: 10.3390/ijms232314795 (PMC9739762; doi:10.3390/ijms232314795)
Supplement: Supplementary file 1 [file ijms-23-14795-s001.zip › ijms-1998495-supplementary.pdf]

Supplementary Material

LAG3/CD4 Genes Variants and the Risk for Restless Legs Syndrome

Félix Javier Jiménez-Jiménez [1,*], Javier Gómez-Tabales [2], Hortensia Alonso-Navarro [1], Christopher Rodríguez [2], Laura Turpín-Fenoll [3], Jorge Millán-Pascual [3], Ignacio Álvarez [4,5], Pau Pastor [4,5,6], Marisol Calleja [1], Rafael García-Ruiz [3], Santiago Navarro-Muñoz [3], Marta Recio-Bermejo [3], José Francisco Plaza-Nieto [1], Esteban García-Albea [7], Elena García-Martín [2] and José A. G. Agúndez [2]

[1] Section of Neurology, Hospital Universitario del Sureste, E28500 Arganda del Rey, Spain
[2] ARADyAL Instituto de Salud Carlos III, University Institute of Molecular Pathology Biomarkers, Universidad de Extremadura, E10003 Cáceres, Spain
[3] Section of Neurology, Hospital La Mancha-Centro, E13600 Alcázar de San Juan, Spain
[4] Fundació per la Recerca Biomèdica i Social Mútua de Terrassa, E08221 Terrassa, Spain
[5] Movement Disorders Unit, Department of Neurology, Hospital Universitari Mutua de Terrassa, E08221 Terrassa, Spain
[6] Unit of Neurodegenerative Diseases, Department of Neurology, The Germans Trias i Pujol Research Institute (IGTP), University Hospital Germans Trias i Pujol, E08916 Badalona, Spain
[7] Department of Medicine-Neurology, Universidad de Alcalá, E28801 Alcalá de Henares, Spain
* Correspondence: fjavier.jimenez@salud.madrid.org or felix.jimenez@sen.es; Tel.: +34-636-968-395

Supplementary Table S1. Genotypes and allelic variants of female patients with RLS and healthy controls. The values in each cell represent the number (percentage; 95% confidence intervals) P: crude probability; Pc: probability after multiple comparisons; NPV: negative predictive value.

**GENOTYPES**	***RLS WOMEN (N=217)***	***CONTROL WOMEN (N=270)***	***INTERGROUP COMPARISON OR (95%CI), P; PC ; NPV (95%CI)***
rs1922452 A/A	39 (18.0; 12.9-23.1)	46 (17.0; 12.6-21.5)	1.07 (0.67-1.71); 0.787; 0.852; 0.56 (0.54-0.58)
rs1922452 A/G	106 (48.8; 42.2-55.5)	126 (46.7; 40.7-52.6)	1.09 (0.76-1.56); 0.632; 0.852; 0.57 (0.52-0.61)
rs1922452 G/G	72 (33.2; 26.9-39.4)	98 (36.3; 30.6-42.0)	0.88 (0.60-1.27); 0.474; 0.852; 0.54 (0.51-0.58)
rs951818 A/A	71 (32.7; 26.5-39.0)	96 (35.6; 29.8-41.3)	0.88 (0.60-1.29); 0.513; 0.852; 0.54 (0.51-0.58)
rs951818 A/C	114 (52.5; 45.9-59.2)	131 (48.5; 42.6-54.5)	1.17 (0.82-1.68); 0.379; 0.852; 0.57 (0.53-0.62)
rs951818 C/C	32 (14.7; 10.0-19.5)	43 (15.9; 11.6-20.3)	0.91 (0.56-1.50); 0.720; 0.852; 0.55 (0.53-0.57)
rs870849 C/C	87 (40.1; 33.6-46.6)	106 (39.3; 33.4-45.1)	1.04 (0.72-1.49); 0.852; 0.852; 0.56 (0.52-0.60)
rs870849 C/T	102 (47.0; 40.4-53.6)	132 (48.9; 42.9-54.9)	0.93 (0.65-1.33); 0.679; 0.852; 0.55 (0.50-0.59)
rs870849 T/T	28 (12.9; 8.4-17.4)	32 (11.9; 8.0-15.7)	1.10 (0.64-1.89); 0.726; 0.852; 0.56 (0.54-0.58)
**ALLELES**	***RLS WOMEN (N= 434 ALLELES)***	***CONTROL WOMEN (N= 540 ALLELES)***	***INTERGROUP COMPARISON OR (95%CI), P; PC ; NPV (95%CI)***
rs1922452 A	184 (42.4; 37.7-47.0)	218 (40.4; 36.2-44.5)	1.09 (0.84-1.41); 0.523; 0.972; 0.56 (0.54-0.59)
rs1922452 G	250 (57.6; 53.0-62.3)	322 (59.6; 55.5-63.8)	0.92 (0.71-1.19); 0.523; 0.972; 0.54 (0.50-0.58)
rs951818 A	256 (59.0; 54.4-63.6)	323 (59.8; 55.7-64.0)	0.97 (0.75-1.25); 0.794; 0.972; 0.55 (0.51-0.59)
rs951818 C	178 (41.0; 36.4-45.6)	217 (40.2; 36.0-44.3)	1.04 (0.80-1.34); 0.794; 0.972; 0.56 (0.53-0.59)
rs870849 C	276 (63.6; 59.1-68.1)	344 (63.7; 59.6-67.8)	1.00 (0.77-1.29); 0.972; 0.972; 0.55 (0.51-0.60)
rs870849 T	158 (36.4; 31.9-40.9)	196 (36.3; 32.2-40.4)	1.00 (0.77-1.29); 0.972; 0.972; 0.55 (0.51-0.60)

Supplementary Table S2. Genotypes and allelic variants of male patients with RLS and healthy controls. The values in each cell represent the number (percentage; 95% confidence intervals) P: crude probability; Pc: probability after multiple comparisons; NPV: negative predictive value.

**GENOTYPES**	***RLS MEN*** ***(N=68)***	***CONTROL MEN*** ***(N=80)***	***INTERGROUP COMPARISON*** ***OR (95%CI) P; PC ; NPV (95%CI)***
rs1922452 A/A	13 (19.1; 9.8-28.5)	13 (16.3; 8.2-24.3)	1.22 (0.52-2.84); 0.649; 0.861; 0.55 (0.51-0.59)
rs1922452 A/G	29 (42.6; 30.9-54.4)	39 (48.8; 37.8-59.7)	0.78 (0.41-1.50); 0.459; 0.861; 0.51 (0.44-0.59)
rs1922452 G/G	26 (38.2; 26.7-49.8)	28 (35.0; 24.5-45.5)	1.15 (0.59-2.25); 0.685; 0.861; 0.55 (0.49-0.62)
rs951818 A/A	28 (41.2; 29.5-52.9)	31 (38.8; 28.1-49.4)	1.11 (0.57-2.14); 0.765; 0.861; 0.55 (0.48-0.62)
rs951818 A/C	30 (44.1; 32.3-55.9)	38 (47.5; 36.6-58.4)	0.87 (0.46-1.67); 0.682; 0.861; 0.53 (0.45-0.61)
rs951818 C/C	10 (14.7; 6.3-23.1)	11 (13.8; 6.2-21.3)	1.08 (0.43-2.73); 0.869; 0.869; 0.54 (0.51-0.58)
rs870849 C/C	33 (48.5; 36.7-60.4)	32 (40.0; 29.3-50.7)	1.41 (0.74-2.72); 0.299; 0.861; 0.58 (0.50-0.65)
rs870849 C/T	26 (38.2; 26.7-49.8)	36 (45.0; 34.1-55.9)	0.76 (0.39-1.46); 0.407; 0.861; 0.51 (0.44-0.59)
rs870849 T/T	9 (13.2; 5.2-21.3)	12 (15.0; 7.2-22.8)	0.86 (0.34-1.20); 0.760; 0.861; 0.54 (0.50-0.57)
**ALLELES**	***RLS MEN*** ***(N= 136 ALLELES)***	***CONTROL MEN*** ***(N= 160 ALLELES)***	***INTERGROUP COMPARISON*** ***OR (95%CI) P; PC ; NPV (95%CI)***
rs1922452 A	55 (40.4; 32.2-48.7)	65 (40.6; 33.0-48.2)	0.99 (0.62-1.58); 0.974; 0.974; 0.54 (0.49-0.59)
rs1922452 G	81 (59.6; 51.3-67.8)	95 (59.4; 51.8-67.0)	1.01 (0.63-1.61); 0.974; 0.974; 0.54 (0.47-0.61)
rs951818 A	86 (63.2; 55.1-71.3)	100 (62.5; 55.0-70.0)	1.03 (0.64-1.66); 0.896; 0.974; 0.55 (0.47-0.62)
rs951818 C	50 (36.8; 28.7-44.9)	60 (37.5; 30.0-45.0)	0.97 (0.60-1.56); 0.896; 0.974; 0.54 (0.49-0.58)
rs870849 C	92 (67.6; 59.8-75.5)	100 (62.5; 55.0-70.0)	1.26 (0.78-2.03); 0.356; 0.974; 0.58 (0.50-0.66)
rs870849 T	44 (32.4; 24.5-40.2)	60 (37.5; 30.0-45.0)	0.78 (0.49-1.29); 0.356; 0.974; 0.52 (0.48-0.57)

Supplementary Table S3. ***G*****enotypes and allelic variants of patients with RLS distributed by response to dopamine agonists. The values in each cell represent the number (percentage; 95% confidence intervals).**

**GENOTYPES**	**POSITIVE RESPONSE TO DOPAMINE AGONISTS (N=222)**	**NEGATIVE RESPONSE TO DOPAMINE AGONISTS (N=18)**	**INTERGROUP COMPARISON VALUES** ***OR (95%CI) P; PC; NPV OR (95%CI)***
rs1922452 A/A	37 (16.7; 11.8-21.6)	4 (22.2; 3.0-41.4)	0.70 (0.22-2.25); 0.548; 0.789; 0.07 (0.05-0.08)
rs1922452 A/G	105 (47.3; 40.7-53.9)	7 (38.9; 16.4-61.4)	1.41 (0.53-3.77); 0.493; 0.789; 0.09 (0.05-0.12)
rs1922452 G/G	80 (36.0; 29.7-42.4)	7 (38.9; 16.4-61.4)	0.89 (0.33-2.37); 0.809; 0.910; 0.07 (0.04-0.10)
rs951818 A/A	85 (38.3; 31.9-44.7)	4 (22.2; 3.0-41.4)	2.17 (0.69-6.19); 0.176; 0.789; 0.09 (0.06-0.11)
rs951818 A/C	109 (49.1; 42.5-55.7)	10 (55.6; 32.6-78.5)	0.77 (0.29-2.03); 0.599; 0.789; 0.07 (0.03-0.10)
rs951818 C/C	28 (12.6; 8.2-17.0)	4 (22.2; 3.0-41.4)	0.51 (0.16-1.64); 0.250; 0.789; 0.07 (0.05-0.08)
rs870849 C/C	100 (45.0; 38.5-51.6)	7 (38.9; 16.4-61.4)	1.29 (0.48-3.45); 0.614; 0.789; 0.08 (0.05-0.11)
rs870849 C/T	97 (43.7; 37.2-50.2)	8 (44.4; 21.5-67.4)	0.97 (0.37-2.55); 0.951; 0.951; 0.07 (0.04-0.10)
rs870849 T/T	25 (11.3; 7.1-15.4)	3 (16.7; -0.6-33.9)	0.64 (0.17-2.35); 0.493; 0.789; 0.07 (0.05-0.08)
**ALLELES**	**POSITIVE RESPONSE TO DOPAMINE AGONISTS (N=444 ALLELES)**	**NEGATIVE RESPONSE TO DOPAMINE AGONISTS (N=36 ALLELES)**	**INTERGROUP COMPARISON VALUES** ***OR (95%CI) P; PC; NPV OR (95%CI)***
rs1922452 A	179 (40.3; 35.8-44.9)	15 (41.7; 25.6-57.8)	0.95 (0.48-1.88); 0.874; 0.874; 0.07 (0.05-0.09)
rs1922452 G	265 (59.7; 55.1-64.2)	21 (58.3; 42.2-74.4)	1.06 (0.53-2.11); 0.874; 0.874; 0.08 (0.05-0.11)
rs951818 A	279 (62.8; 58.3-67.3)	18 (50.0; 33.7-66.3)	1.69 (0.86-3.34); 0.128; 0.384; 0.10 (0.07-0.13)
rs951818 C	165 (37.2; 32.7-41.7)	18 (50.0; 33.7-66.3)	0.59 (0.30-1.17); 0.128; 0.384; 0.06 (0.04-0.08)
rs870849 C	297 (66.9; 62.5-71.3)	22 (61.1; 45.2-77.0)	1.29 (0.64-2.59); 0.480; 0.720; 0.09 (0.05-0.13)
rs870849 T	147 (33.1; 28.7-37.5)	14 (38.9; 23.0-54.8)	0.78 (0.39-1.56); 0.480; 0.720; 0.07 (0.05-0.09)

Supplementary Table S4. *G*enotypes and allelic variants of patients with RLS distributed by response to clonazepam (CNZ). The values in each cell represent the number (percentage; 95% confidence intervals).

**GENOTYPES**	**POSITIVE RESPONSE TO CNZ (N=92)**	**NEGATIVE RESPONSE TO CNZ (N=16)**	**INTERGROUP COMPARISON VALUES** ***OR (95%CI) P; PC; NPV OR (95%CI)***
rs1922452 A/A	22 (23.9; 15.2-32.6)	1 (6.3; -5.6-18.1)	4.71 (0.59-37.74); 0.113; 0.337; 0.18 (0.13-0.19)
rs1922452 A/G	39 (42.4; 32.3-52.5)	6 (37.5; 13.8-61.2)	1.23 (0.41-3.66); 0.715; 0.715; 0.16 (0.10-0.21)
rs1922452 G/G	31 (33.7; 24.0-43.4)	9 (56.3; 31.9-80.6)	0.40 (0.13-1.16); 0.086; 0.337; 0.10 (0.05-0.16)
rs951818 A/A	30 (32.6; 23.0-42.2)	8 (50.0; 25.5-74.5)	0.48 (0.17-1.41); 0.181; 0.337; 0.11 (0.06-0.17)
rs951818 A/C	49 (53.3; 43.1-63.5)	7 (43.8; 19.4-68.1)	1.47 (0.50-4.27); 0.484; 0.622; 0.17 (0.10-0.24)
rs951818 C/C	13 (14.1; 7.0-21.2)	1 (6.3; -5.6-18.1)	2.47 (0.30-20.31); 0.389; 0.584; 0.16 (0.12-0.17)
rs870849 C/C	40 (43.5; 33.3-53.6)	6 (37.5; 13.8-61.2)	1.28 (0.43-3.82); 0.657; 0.715; 0.16 (0.10-0.22)
rs870849 C/T	41 (44.6; 34.4-54.7)	10 (62.5; 38.8-86.2)	0.48 (0.16-1.44); 0.187; 0.337; 0.11 (0.05-0.18)
rs870849 T/T	11 (12.0; 5.3-18.6)	0 (0.0; 0.0-0.0)	1.20 (0.82-1.20) [1]; 0.146; 0.337; 0.17 (0.13-0.17)
**ALLELES**	**POSITIVE RESPONSE TO CNZ (N=184 ALLELES)**	**NEGATIVE RESPONSE TO CNZ (N=32 ALLELES)**	**INTERGROUP COMPARISON VALUES** ***OR (95%CI) P; PC; NPV OR (95%CI)***
rs1922452 A	83 (45.1; 37.9-52.3)	8 (25.0; 10.0-40.0)	2.47 (1.06-5.78); 0.034; 0.102; 0.19 (0.15-022)
rs1922452 G	101 (54.9; 47.7-62.1)	24 (75.0; 60.0-90.0)	0.41 (0.17-0.95); 0.034; 0.102; 0.09 (0.04-0.15)
rs951818 A	109 (59.2; 52.1-66.3)	23 (71.9; 56.3-87.5)	0.57 (0.25-1.30); 0.177; 0.266; 0.11 (0.06-0.17)
rs951818 C	75 (40.8; 33.7-47.9)	9 (28.1; 12.5-43.7)	1.76 (0.77-4.01); 0.177; 0.266; 0.17 (0.13-0.21)
rs870849 C	121 (65.8; 58.9-72.6)	22 (68.8; 52.7-84.8)	0.87 (0.39-1.96); 0.742; 0.742; 0.14 (0.08-0.213)
rs870849 T	63 (34.2; 27.4-41.1)	10 (31.3; 15.2-47.3)	1.15 (0.51-2.57); 0.742; 0.742; 0.15 (0.12-0.19)

Armitage's test for trend with the number of variant rs1922452 alleles = Z- squared = 3.95, P = 0.047. [1] The RR value is shown instead of the OR because at least one of the values in the comparison is equal to 0.

Supplementary Table S5. *G*enotypes and allelic variants of patients with RLS distributed by response to GABAergic drugs. The values in each cell represent the number (percentage; 95% confidence intervals).

**GENOTYPES**	**POSITIVE RESPONSE TO GABAERGIC DRUGS (N=51)**	**NEGATIVE RESPONSE TO GABAERGIC DRUGS (N=12)**	**INTERGROUP COMPARISON VALUES** ***OR (95%CI), P; PC; NPV OR (95%CI)***
rs1922452 A/A	12 (23.5; 11.9-35.2)	2 (16.7; -4.4-37.8)	1.54 (0.30-8.01); 0.610; 0.620; 0.20 (0.14-0.24)
rs1922452 A/G	17 (33.3; 20.4-46.3)	6 (50.0; 21.7-78.3)	0.50 (0.14-1.79); 0.284; 0.535; 0.15 (0.07-0.23)
rs1922452 G/G	22 (43.1; 29.5-56.7)	4 (33.3; 6.7-60.0)	1.52 (0.41-5.69); 0.538; 0.620; 0.22 (0.12-0.29)
rs951818 A/A	18 (35.3; 22.2-48.4)	1 (8.3; -7.3-24.0)	6.00 (0.72-50.30), 0.069; 0.273; 0.25 (0.17-0.27)
rs951818 A/C	22 (43.1; 29.5-56.7)	10 (83.3; 62.2-104.4)	0.15 (0.03-0.76); 0.013; 0.117; 0.07 (0.01-0.18)
rs951818 C/C	11 (21.6; 10.3-32.9)	1 (8.3; -7.3-24.0)	3.03 (0.35-26.05); 0.297; 0.535; 0.22 (0.15-0.23)
rs870849 C/C	21 (41.2; 27.7-54.7)	4 (33.3; 6.7-60.0)	1.40 (0.37-5.26); 0.620; 0.620; 0.21 (0.12-0.28)
rs870849 C/T	26 (51.0; 37.3-64.7)	5 (41.7; 13.8-69.6)	1.46 (0.41-1.20); 0.565; 0.620; 0.22 (0.11-0.31)
rs870849 T/T	4 (7.8; 0.5-15.2)	3 (25.0; 0.5-49.5)	0.26 (0.05-1.34); 0.091; 0.273; 0.16 (0.12-0.20)
**ALLELES**	**POSITIVE RESPONSE TO GABAERGIC DRUGS (N=110 ALLELES)**	**NEGATIVE RESPONSE TO GABAERGIC DRUGS (N=24 ALLELES)**	**INTERGROUP COMPARISON VALUES** ***OR (95%CI), P; PC; NPV OR (95%CI)***
rs1922452 A	41 (40.2; 30.7-49.7)	10 (41.7; 21.9-61.4)	0.94 (0.38-2.32); 0.895; 0.895; 0.19 (0.12-0.24)
rs1922452 G	61 (59.8; 50.3-69.3)	14 (58.3; 38.6-78.1)	1.06 (0.43-2.62); 0.895; 0.895; 0.20 (0.11-0.29)
rs951818 A	58 (56.9; 47.3-66.5)	12 (50.0; 30.0-70.0)	1.32 (0.54-3.21); 0.544; 0.816; 0.21 (0.13-0.30)
rs951818 C	44 (43.1; 33.5-52.7)	12 (50.0; 30.0-70.0)	0.76 (0.31-1.85); 0.544; 0.816; 0.17 (0.11-0.24)
rs870849 C	68 (66.7; 57.5-75.8)	13 (54.2; 34.2-74.1)	1.69 (0.69-4.17); 0.252; 0.756; 0.24 (0.15-0.35)
rs870849 T	34 (33.3; 24.2-42.5)	11 (45.8; 25.9-65.8)	0.59 (0.24-1.46); 0.252; 0.756; 0.16 (0.10-0.22)
